# Tetravalent dengue DNA vaccine is not immunogenic when delivered by retrograde infusion into salivary glands

**DOI:** 10.1186/s40794-020-00111-5

**Published:** 2020-06-03

**Authors:** Guy El Helou, Todd A. Ponzio, Joseph F. Goodman, Maria Blevins, David L. Caudell, Kanakatte S. Raviprakash, Daniel Ewing, Maya Williams, Kevin R. Porter, John W. Sanders

**Affiliations:** 1 Department of Medicine, Division of Infectious Diseases and Global Medicine, University of Florida, Gainesville, FL USA; 2 Department of Medicine, Division of Infectious Diseases, Wake Forest School of Medicine, Winston-Salem, NC USA; 3 Department of Otolaryngology, George Washington School of Medicine and Health Sciences, Washington, DC 20037 USA; 4 Department of Pathology, Section on Comparative Medicine, Wake Forest School of Medicine, Winston-Salem, NC USA; 5 Naval Medical Research Center, Silver Spring, MD USA

**Keywords:** Salivary gland infusion, Tetravalent dengue vaccine, DNA vaccine, Non-human primate

## Abstract

**Introduction and background:**

A tetravalent DNA vaccine for Dengue virus is under development but has not yet achieved optimal immunogenicity. Salivary glands vaccination has been reported efficacious in rodents and dogs. We report on a pilot study testing the salivary gland as a platform for a Dengue DNA vaccine in a non-human primate model.

**Materials and methods:**

Four cynomolgus macaques were used in this study. Each macaque was pre-medicated with atropine and sedated with ketamine. Stensen’s duct papilla was cannulated with a P10 polyethylene tube, linked to a 500ul syringe. On the first two infusions, all macaques were infused with 300ul of TVDV mixed with 2 mg of zinc. For the 3rd infusion, to increase transfection into salivary tissue, two animals received 100uL TVDV mixed with 400uL polyethylenimine 1μg/ml (PEI) and the other two animals received 500uL TVDV with zinc. Antibody titers were assessed 4 weeks following the second and third infusion.

**Results and conclusions:**

SGRI through Stensen’s duct is a well-tolerated, simple and easy to reproduce procedure. TVDV infused into macaques salivary glands elicited a significantly weaker antibody response than with different delivery methods.

## Introduction and background

Dengue is a major arboviral disease that has evaded effective vaccine for over 50 years. The only currently licensed vaccine, Dengvaxia® is not suited for people who have not been infected by dengue before [1]. Traditional methods of vaccination by attenuating the virus or purifying the proteins have not been successful [2] DNA vaccines have long been known to produce good immunogenicity in different animal models [3, 4]. A tetravalent DNA vaccine for Dengue virus (TVDV) is under development but has not yet achieved optimal immunogenicity [5]. Alternative administration methods have recently shown promise in boosting the vaccine’s immunogenicity, but further increasing immunogenicity would aid in the vaccine’s development [6]. One lesser-known DNA vaccine administration site reported to result in high titer responses in rodents and dogs is the salivary gland [7–9]. Here, we report on a pilot study testing the feasibility and efficacy of the salivary gland as a platform for a Dengue DNA vaccine in a non-human primate model.

## Materials and methods

The study involved four female cynomolgus monkeys. All were tested for dengue and other flavivirus antibody titers prior to vaccine infusions and all were negative. Twenty minutes prior to performing the procedure, each of the animals was pre-medicated with subcutaneous atropine (0.05 mg/kg) to reduce salivary secretions. Anesthesia and sedation were achieved through intramuscular delivery of ketamine/midazolam (10–15 mg/kg of ketamine and 0.01–0.05 mg/kg of midazolam). The mouth was braced open with the help of standard oral retractors, allowing for access to the sides of the oral cavity. Using standard surgical loops for magnification, the parotid papilla could be easily identified along the buccal pouch facing the second upper molar on each side. The papilla was then dilated with a conical dilator allowing cannulation with size 10 polyethylene tube (PE10) into Stensen’s duct. One end of the tubing was connected to a 500ul syringe. The tubing was gently inserted approximately 2-4 cm into the duct. To prevent back-flow of the infusate, following the duct cannulation, a small amount of cyanoacrylate was placed at the duct opening.

The DNA vaccine is composed of four plasmids, with each plasmid encoding for the pre-membrane and envelope proteins of dengue 1, 2, 3 and 4 [10]. Vaccine infusions were performed on days 0, 28, and 98. Infusions occurred at a rate of 100ul/min. The tube was left in place 5 min after infusion completion to prevent recoiling of the glands tissue with expulsion of the infusate. Food and water was restricted for 2 hours following procedure to reduce salivary secretions. The syringes were prefilled with 300ul of TVDV mixed with 2 mg of zinc. For the third infusion (day 98), in an effort to increase transfection into salivary tissue, two animals received 100ul TVDV mixed with 400ul polyethylenamine (PEI) 1μg/ml for total volume of 500ul/gland; and the other two macaques were infused with 500ul/gland TVDV solution with zinc. Serial antibody titers were obtained 4 weeks following second and third infusion as previously described [6]. Thereafter, we went on to test whether or not the procedure resulted in delivery of the infusate into the gland as planned; and whether or not transfection of the infused DNA occurred in the salivary gland cells.

To ensure our methods resulted in delivery of the infusate into the parotids, we performed fluoroscopy using contrast imaging. Additionally, to test whether or not plasmids were able to efficiently transduce cynomolgus monkey salivary gland cells, plasmid DNA encoding for Enhanced Green Fluorescent Protein (EGFP) was infused. This was compared with an Adenovirus (serotype 5, Ad5) that coded for EGFP, as Ad5 is reported to efficiently transduce both acinar and ductal salivary gland cells for both rhesus macaques, as well as humans [11, 12].

## Results

**Fig. 1:**
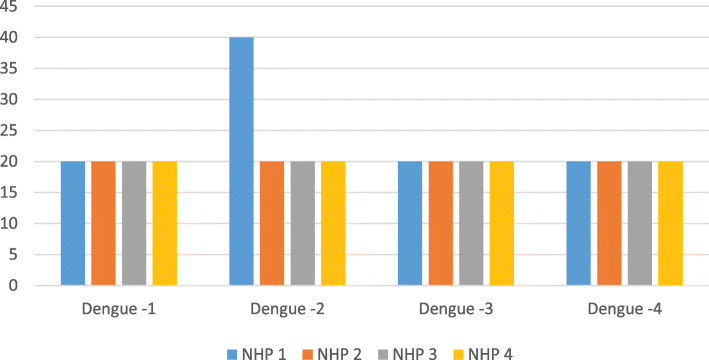
Dengue antibody titers on day 30 by serotype in the 4 NHPs

**Fig. 2:**
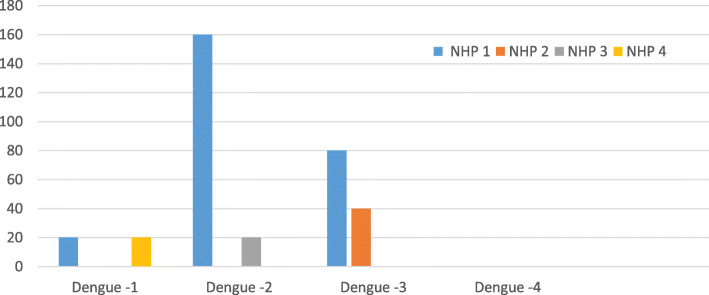
Dengue antibody titers on day 100 by serotype in the 4 NHPs. NHP 1 and NHP 2 received PEI formulated vaccine

The procedure was well tolerated, simple and easy to reproduce. However, it resulted in poor immunological responses. Anti-Dengue Immunoglobulin G (IGG) antibody titers did not pass the threshold value of 1:20 (mean dilution) after two doses of TVDV plus zinc. Adding PEI to the infusate in two animals (NHP 1, 2), resulted in only slightly better neutralizing antibody titers for NHP 1, who had dengue serotype 2 titers going up to 1:160 (Figs. 1 and 2).

**Fig. 3:**
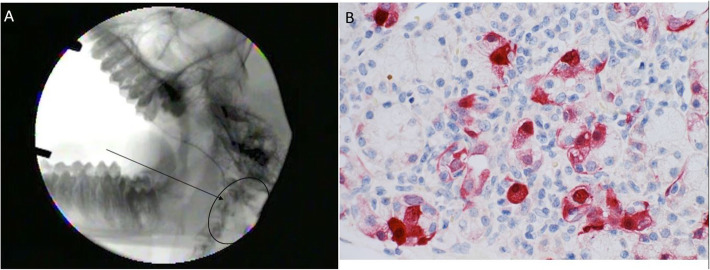
Fluoroscopy and Ad5 salivary glands infusions. Panel **a**: Please note adequate distribution of radiocontrast into Stensen’s duct and parotid tissue following retrograde salivary gland infusion. Panel **b**: note the transfection of both acinar and ductal cells by Ad5 vectored Enhanced Green Fluorescent Protein detected by anti-GFP (red stain)

**Fig. 4:**
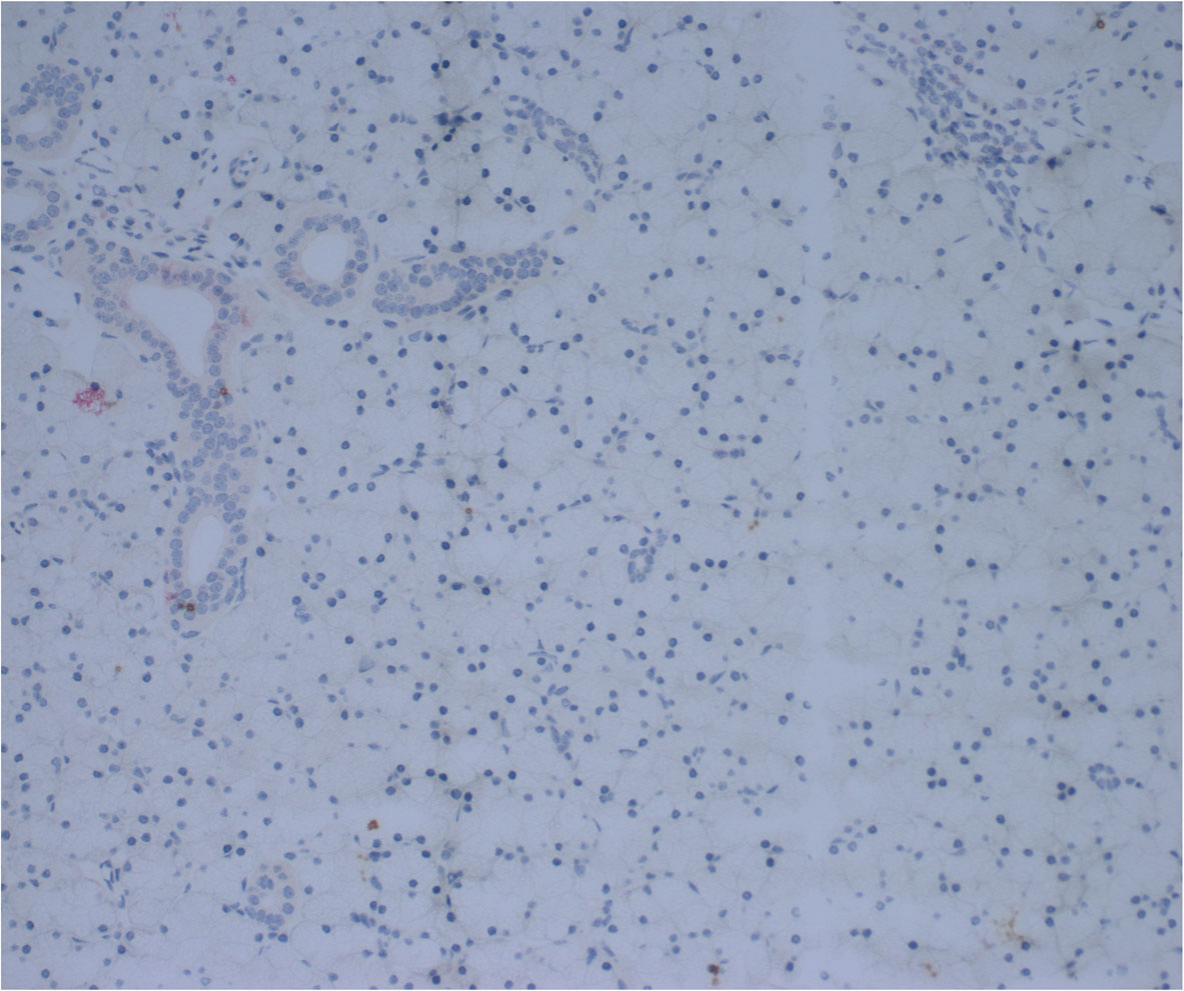
Pathology slides at 20x magnification of parotid glands infused with plasmid vectored EGFP. Note absence of expression of EGFP (no red stain) in parotid tissue infused with plasmid vectored EGFP

As shown in Fig. 3, not only did the fluoroscopy confirm that the procedure resulted in the infusate filling the targeted parotid gland, the presence of EGFP was detected by immunohistochemistry in the glands infused with the Ad5 (Fig. 3a, b). However, no EGFP could be detected in the salivary glands treated with EGFP plasmid DNA (Fig. 4).

## Discussion

A number of new approaches are being explored to enhance immunologic responses to DNA vaccines including identifying better vectors, use of molecular adjuvants, electroporation, and delivery into tissue spaces rich in antigen presenting cells. Vaccines and vaccine candidates that elicit sub-optimal immune responses are natural test candidates for these types of approaches and technologies. For example, a recent study that assessed utilizing electroporation to administer TVDV showed encouraging results [6]. Infusion of plasmid DNA into the salivary gland has been reported to elicit a robust immune response in both rats and dogs [8, 9]. Here, we report on a pilot study testing this approach in cynomolgus macaques and find that those earlier findings could not be validated in this particular non-human primate model.

While the method of parotid gland infusion was simple and reproducible and resulted in no complications with the subject animals, infusion of the plasmid vaccine did not elicit an appreciable immune response. These findings appeared at odds with the sizable titers reported by Tucker et al. in response to DNA vaccine delivery into the salivary glands of rodents and dogs [8, 9] Given the better immunologic responses to this vaccine reported using standard techniques in this model [6] and the exploratory nature of this approach, it was important to confirm that the infusion technique was adequate to deliver the vaccine to the target tissue. We repeated the procedure under fluoroscopic imaging and demonstrated that a similar volume, fluid bolus was appropriately distributed throughout the gland, confirming the validity of the delivery technique. To determine if the delivered plasmid vector was efficiently transfected, we compared the uptake of a plasmid and an Ad5 vector coding for EGFP. EGFP was readily visualized throughout the gland following Ad5 infusion (see Fig. 3b), but no EGFP was seen in response to EGFP plasmid infusion (Fig. 4). Importantly, the Ad5 vector experiment and immunohistochemical confirmation of EGFP in the gland not only validates previous work demonstrating Ad5 transduction of salivary gland cells in rhesus macaques and humans, but extends that work into the cynomolgus model as well [13, 14].

To our knowledge, this is the first study to test DNA vaccination though the salivary gland in a non-human primate model. Prior to this, all previous studies pursuing this approach have used mice, rats or dogs, posing a natural question as to whether or not the earlier reported results can be extended into primates [15]. The results reported here collectively indicate there is exceedingly poor transfection of plasmid into salivary gland cells of the cynomolgus macaque. This was found to be the case both in the presence of zinc as well as PEI, casting doubt on this approach being translatable to humans.

If efficient gene transfer to salivary gland cells were to be achieved, this approach would seem worth revisiting. Although Ad5 appears to be an efficient vector, demonstrating tropism for both acinar and ductal cells, the evoked immune response against the vector itself would preclude repeat dosing [16]. However, one research effort has looked to ultrasound assisted gene transfer to enhance plasmid transduction, and has shown promise in both rodents and miniature pigs [17, 18]. For viral vectors to be attractive conduits, the vector should be both efficient at transducing cells and also elicit no (or only a muted) immune response. Toward this end, adeno-associated viruses have been tested, including in non-human primates, though the serotypes tested thus far do not appear capable of transducing acinar cells [19]. Lentivirus appears to transduce both acinar and ductal cells in the rodent model [20] and should be further evaluated in the primate model.

## Conclusion

From a feasibility standpoint, retrograde salivary gland infusion into the parotids was simple and reproducible and could potentially be administered by a primary care provider or a dentist after appropriate training for vaccines where its efficacy would be proven.

This study was designed to build off of previous reports noting robust systemic immune responses following the delivery of plasmid DNA with zinc or lipid directly through the salivary duct. While the method is reported to work in rodents and dogs and result in a strong immune response, direct infusion of naked plasmid in the presence of zinc or PEI does not result in a notable immune response in non-human primates. Future studies should consider including augmenting technologies, such as ultrasound assisted gene transfer, or using alternative gene transfer approaches.

## Data Availability

The datasets used and/or analyzed during the current study are available from the corresponding author on reasonable request.
